# Can data assetization drive high-quality enterprise development?—Evidence from China’s “Specialized, refined, unique, and innovative” SMEs

**DOI:** 10.1371/journal.pone.0335903

**Published:** 2025-11-07

**Authors:** Lin Li, Jiulin Zhu

**Affiliations:** College of Economic and Management, Heilongjiang Bayi Agricultural University, Daqing, China; State University of Bangladesh, BANGLADESH

## Abstract

Data assetization empowers the high-quality development of “Specialized, Refined, Distinctive, and Innovative” (SRDI) small and medium-sized enterprises (SMEs) by enhancing organizational performance and driving innovation. Based on this, this study selects SRDI SMEs in China from 2013 to 2023 as samples. It constructs a keyword graph spectrum based on enterprise annual report texts to quantitatively assess the level of data assetization and investigates whether data assetization can facilitate the high-quality development of these SMEs. The research findings indicate that: (1) data assetization significantly contributes to the high-quality development of SRDI SMEs, primarily through two mechanisms—enhancing strategic differentiation and improving resource allocation efficiency; (2) the robustness of these findings is confirmed through a series of tests, including alternative specifications of dependent variables, inclusion of additional control variables, subsample analyses, and the exclusion of potential confounding factors; (3) further analysis grounded in the TOE (Technology–Organization–Environment) framework demonstrates that the positive impact of data assetization is amplified by firm-level innovation capacity, customer relationship strength, regional characteristics, and the extent of enterprise digital finance development. This study contributes to advancing the understanding of how data assetization influences the high-quality development of SRDI SMEs, offering a theoretical foundation for future research in this domain.

## 1. Introduction

The high-quality development of “specialized, refined, distinctive, and innovative” (SRDI) enterprises serves as a pivotal driver in advancing the real economy. In 2024, China formally introduced a policy explicitly supporting the development of such enterprises, thereby providing strategic guidance for enhancing the growth of small and medium-sized enterprises (SMEs) with specialized capabilities. Although the specific concept of SRDI enterprises is unique to China and lacks a direct international equivalent, global initiatives—including the European Union’s “Competitiveness Compass” strategy launched in early 2025, the establishment of the “Global Innovation Center” in Bangalore, India, and financial support from the European Investment Fund (EIF) for innovative SMEs in Central and Eastern Europe—demonstrate a shared international commitment to fostering innovation-driven enterprises. Despite being in a period of favorable policy incentives and industrial upgrading opportunities, most of these enterprises continue to face significant survival pressures. The key to their sustainable development lies in effectively enhancing total factor productivity, which fundamentally requires establishing dynamic competitive advantages through strategic differentiation and optimal resource allocation. Amidst the global wave of digital transformation, data has emerged as a pivotal production factor [1]. The process of data assetization offers a transformative paradigm for enterprise development. The “Interim Provisions on Accounting Treatment of Enterprise Data Resources”, issued by China’s Ministry of Finance, marks the first official establishment of accounting standards recognizing data resources as a distinct asset class. By introducing a dual recognition framework—under both intangible assets and inventory—the provisions enable the valuation and activation of enterprises’ dormant data assets [2], thereby laying a critical institutional and legal foundation for corporate data assetization. Furthermore, the 2025 Government Work Report outlines a three-phase strategy for advancing the market-oriented reform of data elements, underscoring the pivotal role of data as a production factor in driving high-quality economic development [3]. Data assetization represents a critical pathway for unlocking the value of data elements, defined as the systematic process of transforming raw data resources into economically valuable assets for effective management and operational utilization. This article distinguishes between four concepts: digitalization, digital assets, data assetization, and data capitalization. Digital assets is a noun, while digitalization, data assetization, and data capitalization are verbs, indicating a process. The relationship among these four is progressive, specifically manifested as: digitalization → formation of digital assets → transformation into formal assets through data assetization → ultimately creation of cash flow and wealth through data capitalization. This process involves the structured collection, processing, analysis, and application of data to convert intangible inputs into formal, measurable assets recognized on an enterprise’s balance sheet. In contrast to traditional asset ownership models, the rights structure associated with data assets is inherently more complex [4].Organizations must therefore establish a coherent “data resources–data assetization–data assets” conceptual framework [5] and strategically integrate data assetization into investment planning and resource allocation processes. Such integration enables enterprises to enhance cost efficiency, improve operational performance, generate new revenue streams [6], and support sustainable, high-quality development.

Currently, a growing body of academic research has focused on data assetization, with scholarly inquiry primarily centered on two key dimensions: the mechanisms underlying its implementation and its economic implications. First, regarding the implementation framework, the pathway of data assetization is commonly structured as a sequential triad—data rights confirmation, data valuation, and data circulation—each exerting distinct influences on enterprise-level high-quality development. On the issue of data rights confirmation, some researchers regard it as a foundational prerequisite for data assetization, arguing that enterprises, through continuous data collection, analysis, and integration, develop de facto control over data and thus should be granted corresponding ownership rights [7]. In contrast, other studies adopt a value chain perspective, contending that data ownership should not be exclusively assigned to enterprises but must also recognize the contributions of individual users and other stakeholders involved in value creation across the chain [8]. With respect to data valuation, effective valuation mechanisms enhance the transparency and interpretability of data assets, enabling organizations to respond more dynamically to market changes, foster business model innovation, and ultimately advance sustainable, high-quality development [9].Furthermore, concerning data circulation, the effective implementation of this process facilitates the transformation of data into consumable, tradable, and transferable assetized commodities [10], thereby optimizing operational management, generating measurable economic value for enterprises, and reinforcing their developmental capacity. Second, from the standpoint of economic effects, data assetization exerts a dual-driven impact. On one hand, it promotes the advancement of new-quality productive forces by broadening enterprise credit enhancement mechanisms, thereby improving innovation performance and cultivating new drivers for sustainable, high-quality development [11]. On the other hand, it gives rise to a demand-oriented intelligent supply chain model, driving innovation in consumption patterns and further strengthening enterprise-level developmental quality [12].

Based on a comprehensive review of the existing literature, this study identifies the following research questions: As a key mechanism for realizing the value of data elements, can data assetization drive the high-quality development of micro-level enterprises? What are the underlying mechanisms governing this relationship? Focusing on listed specialized and innovative enterprises, we employ text mining techniques to quantify data assetization levels. Our analysis examines the mediating roles of strategic differentiation and resource allocation efficiency, constructing a comprehensive micro-level evidence chain that elucidates how data assetization drives enterprise development.

The key contributions of this paper are as follows: First, prior studies on the empirical analysis of how data assetization empowers the high-quality development of enterprises exhibit certain limitations. This study focuses on SRDI enterprises, offering robust empirical evidence for research in this domain. Second, it examines the underlying mechanisms through which data assetization drives enterprise high-quality development from the perspectives of strategic differentiation and resource allocation efficiency. This not only enhances our understanding of the empowerment mechanism of data assetization but also provides valuable insights for promoting sustainable enterprise development. Third, existing studies predominantly use large enterprises as samples, failing to account for the characteristics of specialized, refined, distinctive, and innovative SMEs—namely, their limited resources and high dependence on innovation. This study fills these gaps through analysis, enriching the research on the impact of data assetization on the high-quality development of “specialized, refined, distinctive, and innovative” (SRDI) small and medium-sized enterprises (SMEs).

## 2. Theoretical analysis and research hypotheses

Data assetization enhances enterprise competitiveness by generating economic value, improving performance, and fostering innovation, thereby facilitating high-quality enterprise development. Extensive empirical research and theoretical analysis indicate that data assetization can elevate enterprise performance through strategic decision-making and empower enterprises with new productive capabilities by optimizing resource allocation [13]. From the two core dimensions of enterprise strategic positioning and operational efficiency, data assetization elucidates the value creation mechanisms of strategic differentiation and resource allocation efficiency. It further systematizes the dual-path synergy mechanism where data assetization transforms into core competitiveness by leveraging strategic differentiation to attract user-generated data, enhancing resource allocation efficiency, and enabling efficiency-driven feedback loops to reinforce and innovate strategic differentiation. Based on this, this paper examines the influence of data assetization on enterprise high-quality development from the perspectives of strategic differentiation and resource allocation efficiency.

### 2.1. Strategic differentiation degree

Traditional SMEs frequently encounter the challenge of the “imitator strategy”, characterized by a lack of clear strategic direction and a tendency toward passive imitation, which hinders their ability to achieve breakthrough growth and generate sustainable competitive advantage. In this context, firms should pursue a deliberate degree of strategic differentiation that diverges from industry norms, thereby transcending the limitations imposed by conventional development models [14, 15]. Data assetization offers enterprises essential data support and advanced analytical capabilities to comprehend and operationalize strategic differentiation. This enables the development of a distinctive strategic differentiation framework. When an enterprise establishes capabilities that exceed industry averages in risk management, data integration, and innovation, strategic differentiation shifts from a cost-bearing function to a core source of competitive advantage. This transformation drives the realization of a value-creation cycle, bridging enhanced operational efficiency with sustainable high-quality development. The analysis is as follows:

First, from the perspective of enterprise risk response capability, data assetization enables the systematic integration of internal and external data resources, transforming them into actionable business insights. This facilitates the dynamic optimization of strategic decision-making and drives innovation in risk prevention and control systems, thereby enhancing the firm’s capacity for risk-taking and organizational resilience [16]. As a result, systemic risks can be more effectively mitigated, supporting the enterprise’s long-term sustainable development. Second, from the customer perspective, data assetization-driven strategic distinctiveness enables the integration of customer data resources across the upstream and downstream segments of the industrial chain. Through the consolidation and analysis of these large-scale datasets, SMEs can develop distinctive market strategies grounded in consumer behavior analytics and supply chain optimization. This capability allows firms to precisely identify market trends and customer needs, reduce information asymmetry between supply and demand, enhance customer satisfaction, and improve the efficiency of production and service delivery—ultimately elevating overall enterprise performance and operational quality. Third, from the innovation perspective, enterprises with high strategic differentiation are more likely to invest in research and development (R&D) and product innovation to establish technological barriers. Among these, SRDI enterprises exhibit a pronounced innovation engine effect, boosting the market competitiveness and green innovation output of SRDI SMEs [17], thus achieving high-quality development. It is evident that through strategic differentiation, enterprises can shift strategic decision-making from passive imitation to active construction and transform risk prevention and control from post-event remediation to pre-event prevention, achieving high-quality development in terms of enterprise risk response, customer satisfaction, and green innovation.

### 2.2. Efficiency of resource allocation

Enterprises can optimize resource allocation and achieve significant economic benefits as well as value multiplication through data algorithm-driven approaches [18], thereby enhancing enterprise performance. Data assetization clarifies the logical framework for improving resource allocation by systematically collecting, analyzing, and processing data resources. It overcomes the informational constraints inherent in traditional resource management, reconfigures the mechanisms of resource value creation, and reshapes the enterprise resource collaboration network. This enables SMEs to transcend the “resource curse”, enhance resource allocation efficiency, and transition from a resource-intensive model to a data-driven paradigm—ultimately facilitating sustainable and high-quality enterprise development [19]. The analysis is as follows:

First, from the perspective of information-enabled decision-making, data assetization—through systematic data governance—transforms data resources into actionable decision-support inputs, thereby enhancing organizational dynamic adjustment capabilities. This enhanced capability enables firms to comprehensively improve resource allocation efficiency by addressing the key limitations of traditional models, namely information asymmetry, latency, and data fragmentation, thus reducing the likelihood of resource misallocation [20]. Second, from the perspective of enterprise value creation, data assetization fundamentally reconfigures the data resource-driven value creation system, enabling SRDI SMEs to shift their resource allocation from experience-based decision-making to data-driven strategies, and from localized optimization to system-wide optimal outcomes. This transformation not only improves firm performance but also empowers SRI enterprises to overcome the constraints of traditional resource rigidity. As a result, firms can evolve from “peripheral participants” into “ecosystem architects”, fostering novel value creation models, accelerating organizational structural upgrading, and contributing to the high-quality development of the real economy [21]. Third, from the perspective of enterprise collaborative linkage, data assetization enhances the integration of inter-firm networks by leveraging connectivity advantages and sustaining long-term governance stability, thereby supporting the high-quality development of SRDI SMEs [22].Thus, through enhanced resource allocation efficiency, SRDI enterprises can achieve high-quality development in terms of information-based decision-making, value creation, and collaborative linkage.

Based on the above theoretical analysis, this paper proposes the following hypotheses:

H1: Data assetization can empower the high-quality development of “specialized, refined, distinctive, and innovative” small and medium-sized enterprises.

H1a: Data assetization can empower the high-quality development of “specialized, refined, distinctive, and innovative” small and medium-sized enterprises by enhancing strategic distinctiveness.

H1b: Data assetization can empower the high-quality development of “specialized, refined, distinctive, and innovative” small and medium-sized enterprises by optimizing resource allocation efficiency.

## 3. Research methods

### 3.1. Sample selection and data sources

This study selects the five batches of SRDI SMEs recognized by China’s Ministry of Industry and Information Technology from 2013 to 2023 as research samples. Subsequently, the following data processing steps were implemented: The following data processing steps are applied: ST and *ST companies, financial and insurance firms, and enterprises with missing key variables or abnormal data are excluded. Subsequently, a 1% and 99% winsorization is performed on each continuous variable to address potential outliers. Ultimately, this process yields 1,670 valid observations. The data utilized in this study are sourced from authoritative databases, including CSMAR, Wind Economic Database, and CNRDS.

### 3.2. Variable selection

#### 3.2.1. Dependent variable.

In this study, the dependent variable is the high-quality development of enterprises, measured by total factor productivity (TFP). The OP method has been found to have limitations in addressing missing data issues; Therefore, the LP method is employed to calculate enterprise-level total factor productivity, which serves as a proxy for estimating the level of economic high-quality development [23].

#### 3.2.2. Core explanatory variable.

The core explanatory variable in this study is the degree of enterprise data assetization (DA). In the academic literature, enterprise data assets are primarily measured using financial data and text analysis methods. Building on prior research, this study adopts an advanced text analysis approach. Specifically, it uses relevant laws and regulations on data assets as the corpus, counts the frequency of 221 data asset-related terms, and employs “information”, “network”, “digital”, and “data” as seed words. Leveraging the Word2Vec neural network model and deep learning techniques, a set of similar words is constructed to measure the enterprise data asset index. Data assetization refers to the process by which enterprises convert data resources into data assets. Based on this concept, the dynamic process of data assetization is quantified and categorized into self-used data assets (ODA) and transactional data assets (DDA) according to their specific applications [24].

#### 3.2.3. Control variables.

Drawing on existing research [25], the control variables selected include enterprise scale (Size), debt-to-asset ratio (LEV), return on total assets (ROA), growth potential (Growth), net cash flow (Cashflow), and enterprise age (Age). These variables are incorporated into the analysis as controls. Additionally, industry (Industry) and year (Year) fixed effects are included to account for potential confounding factors. Specific definitions of all variables are provided in Table 1.

**Table 1:** Main variables and definitions.

Variable Name	Variable Symbol	Variable Definition
**High-quality development of enterprises**	TFP	Total factor productivity calculated based on the LP method
**Degree of enterprise data assetization**	DA	Enterprise data assets measured by text analysis methods
**Self-use data assets**	ODA	ln(1 + frequency of internally utilized data assets)
**Transactional data assets**	DDA	ln(1 + frequency of transactional data assets)
**Enterprise scale**	Size	Natural logarithm of total assets at the end of the year
**Debt-to-asset ratio**	LEV	Total liabilities at the end of the year/ Total assets at the end of the year
**Return on total assets**	ROA	Net profit/ Total assets
**Growth**	Growth	Current year’s operating income/ Last year’s operating income – 1
**Net cash flow**	Cashflow	Net cash flow from operating activities/ Total assets
**Enterprise age**	Age	Number of years since establishment
**Industry fixed effect**	Industry	Industry dummy variable
**Year fixed effect**	Year	Year dummy variable

### 3.3. Model construction

To test Hypothesis 1, this paper develops Model (1) to empirically examine the influence of enterprise data assetization on the high-quality development of SRDI SMEs.


$$TFP_{i,t}=\alpha_{0}+\alpha_{1}DA_{i,t}\alpha_{i}Control_{st}+\Sigma_{Ind}+\Sigma_{i,t}$$ (1)

Among these, the dependent variable $TFP_{i,t}$ denotes the level of high-quality enterprise development, reflecting the enterprise’s high-quality status in period t. The core explanatory variable $DA_{i,t}$ indicates the degree of enterprise data assetization. Here, the subscript i refers to enterprises, and t represents time. Controls signify control variables, and $\Sigma_{i,t}$ is the random disturbance term. To more comprehensively investigate the impact of different types of data assets on enterprise high-quality development, this paper substitutes ODA (self-used data assets) and DDA (transactional data assets) for DA in equation (1) for regression analysis.

Based on the theoretical analysis, data assetization enhances the high-quality development of SRDI enterprises through strategic differentiation and resource allocation efficiency. To test Hypothesis 2 and Hypothesis 3, the influence of the mechanisms of strategic differentiation and resource allocation efficiency is examined by adopting the following method [26]. The specific model setup is as follows:


$$Strategy_{i,t}=\alpha_{0}+\alpha_{1}DA_{i,t}+\alpha_{i}Control_{st}+\Sigma_{Year}+\Sigma_{Ind}+\Sigma_{i,t}$$ (2)

$$Ineff_{i,t}=\alpha_{0}+\alpha_{1}DA_{i,t}+\alpha_{i}Control_{st}+\Sigma_{Year}+\Sigma_{Ind}+\Sigma_{i,t}$$ (3)

Among these, Strategy and Ineff are the dependent variables, representing the strategic distinctiveness and resource allocation efficiency of enterprises in period t, respectively. Following the methodology of Gu et al. to measure firm-level strategic distinctiveness, we first computed resource allocation across six key dimensions: advertising and promotional intensity (sales expenses/revenue), R&D intensity (net intangible assets/revenue), capital intensity (fixed assets/number of employees), fixed asset renewal rate (net value of fixed assets/original value of fixed assets), administrative expense intensity (management expenses/revenue), and financial leverage ((short-term debt + long-term debt + bonds payable)/book value of equity) [27]. Next, for each firm, we calculated the deviation of its strategic indicators from the industry-year mean by subtracting the corresponding industry average and dividing the result by the cross-sectional standard deviation, thereby standardizing the variables; the absolute value was then taken to capture the magnitude of deviation in each strategic dimension. Finally, the standardized absolute deviations across the six dimensions were averaged to construct the strategic distinctiveness index (Strategy). A higher value of this index indicates a greater degree of divergence in strategic positioning between the firm and its industry peers in the same year.

To calculate Ineff, we employ the methodology developed by Richardson (2006) [28] to estimate Equation (4). First, the expected level of investment for firms in the current year is derived based on firm characteristics and economic conditions. The absolute value of the model’s residual is then used as a proxy for Ineff, capturing deviations from optimal investment. A higher value indicates greater inefficiency in corporate resource allocation.


$$\begin{array}{c} Invest_{nit}=\beta_{0}+\beta_{1}Growth_{nit-1}+\beta_{2}Lev_{nit-1}+\beta_{3}Roa_{nit-1}+\beta_{4}Age_{nit-1}+\beta_{5}Size_{nit-1}\\ +\beta_{6}I\mathrm{\backslash nolimits}nvest_{nit-1}+\Sigma_{Indu}+\Sigma_{Year}+\varepsilon_{nit} \end{array}$$ (4)

Among these, the fixed asset investment variable Investnit represents the ratio of the original value of fixed assets to total assets at the beginning of the period; Inevestnit−1 denotes the firm’s fixed asset investment in year t−1, calculated using the same method as above. The remaining variables in the equations are control variables included in this study, while and represent industry and year dummy variables, respectively. The remaining variables in Models (2) and (3) are identical to those in Model (1).

## 4. Empirical analysis

### 4.1. Descriptive statistics

Based on the descriptive statistics presented in Table 2, the key variables exhibit the following characteristics. In terms of high-quality enterprise development (TFP), the observed values range from 4.12 to 10.6, with a mean of 6.4323 and a standard deviation of 0.663. These data features suggest that most enterprises prioritize their own high-quality development, and the overall trend of high-quality enterprise development is steadily and sustainably improving. This provides a micro-level foundation for the transformation and upgrading of the macroeconomy. Regarding the degree of enterprise data assetization (DA), the sample data indicate a minimum value of 0 and a maximum value of 5.26, with a standard deviation of 1.174. This highlights significant variation in data assetization levels among SRDI enterprises, which warrants further exploration. The statistical distributions of the remaining control variables align with expectations, featuring reasonable ranges between the maximum and minimum values. This confirms that the research data are of high quality and reliable.

**Table 2:** Descriptive statistical results.

Variable Name	Mean	Standard Deviation	Median	Minimum	Maximum
**TFP**	6.4323	0.663	6.38	4.12	10.60
**DA**	1.8050	1.174	1.79	0.00	5.26
**ODA**	1.7140	1.143	1.61	0.00	4.89
**DDA**	0.4564	0.794	0.00	0.00	4.01
**Strategy**	0.6403	0.344	0.55	0.20	3.87
**Ineff**	0.1016	0.101	0.07	0.00	0.61
**Size**	21.5389	0.800	21.46	19.65	24.33
**Lev**	0.3398	0.185	0.32	0.03	1.06
**ROA**	0.0367	0.073	0.04	−0.80	0.36
**Growth**	0.2593	0.939	0.08	−1.84	27.27
**CashFlow**	0.0441	0.068	0.04	−0.27	0.33
**Age**	2.9668	0.277	3.00	2.20	3.64

### 4.2. Benchmark regression analysis

#### 4.2.1. Data assetization and the high-quality development of specialized, refined, unique, and innovative enterprises.

The estimation results of Model (1) are presented in Table 3. Column (1) shows that the regression coefficient of DA is positive and statistically significant at the 1% level, indicating that a one-unit increase in enterprise data assetization is associated with a 0.2821-unit increase in the level of high-quality development, thus supporting Hypothesis H1. To further investigate the impact of data assetization on the high-quality development of SRDI SMEs, Columns (2) and (3) present separate regression results for ODA and DDA. The coefficients of both ODA and DDA are positive and significant at the 1% level, suggesting that both self-used data assets and transactional data assets contribute significantly to the high-quality development of SRDI firms. Notably, the coefficient on ODA is larger than that on DDA, implying a relatively stronger positive effect of self-used data assets. This difference may be attributed to the fact that self-used data assets directly capture the core processes and capabilities underlying enterprise value creation—making them a driver (“cause”) of high-quality development. In contrast, transactional data assets primarily reflect the outcomes of value creation and thus serve more as an outcome indicator (“effect”) of such development.

**Table 3:** Benchmark regression results.

	(1)	(2)	(3)
	TFP	TFP	TFP
**DA**	0.2821***		
	(5.2341)		
**ODA**		0.2849***	
		(5.1733)	
**DDA**			0.2032***
			(2.6087)
**Size**	0.8716***	0.8728***	0.8891***
	(12.3555)	(12.3640)	(12.5111)
**Lev**	3.1077***	3.1110***	3.1238***
	(9.0483)	(9.0584)	(9.0609)
**ROA**	−0.8813	−0.9300	−1.0679
	(−1.0574)	(−1.1104)	(−1.2886)
**Growth**	−0.2247**	−0.2221**	−0.1830**
	(−2.5306)	(−2.5064)	(−2.0450)
**Cashflow**	4.8695***	4.8817***	4.9203***
	(5.0659)	(5.0712)	(5.1140)
**Age**	1.4052***	1.4132***	1.4551***
	(6.4222)	(6.4980)	(6.5744)
**_cons**	−18.1459***	−18.1931***	−18.3040***
	(−10.8954)	(−10.9042)	(−10.8287)
**Year fe**	Yes	Yes	Yes
**industry fe**	Yes	Yes	Yes
**N**	1670	1670	1670
**adj. R2**	0.2500	0.2500	0.2415

*, **, and *** indicate significance at the 10%, 5%, and 1% confidence levels respectively. The same applies below.

Regarding the control variables, the significantly positive coefficient of Size reflects that larger enterprises tend to exhibit higher production efficiency and better levels of high-quality development. The significantly positive coefficient of Growth indicates that enterprises with strong growth potential can leverage their capabilities to promote high-quality development. The significantly positive coefficient of Cashflow suggests that enterprises with higher net cash flow possess stronger financial health and investment expansion capabilities, facilitating their ability to achieve high-quality development. Additionally, the significantly positive coefficient of Age implies that enterprises with longer operating periods have a competitive advantage in high-quality development. Overall, the estimation results of the control variables align with the research expectations.

#### 4.2.2. The mechanism of data assetization and the high-quality development of SRDI SMEs.

This paper further examines the mechanism underlying the relationship between data assetization and the high-quality development of SRDI SMEs. The regression analyses were performed by separately substituting DA with ODA and DDA. The results of the mechanism tests are presented in Tables 4 and 5, respectively. To examine the mediating effects of strategic differentiation (Strategy) and resource allocation efficiency (Ineff) on the relationship between data assetization (DA) and high-quality development (TFP), we employed the Bootstrap method. The specific steps are as follows:

**Table 4:** Mechanism test: Strategic difference degree.

Variable	(1)	(2)	(3)	(4)	(5)	(6)	(7)	(8)	(9)
	TFP	Strategy	TFP	TFP	Strategy	TFP	TFP	Strategy	TFP
DA	0.2821***	0.0226***	0.0510***						
	(5.2341)	(2.7558)	(3.3892)						
ODA				0.2849***	0.0211**	0.0559***			
				(5.1733)	(2.5726)	(3.5848)			
DDA							0.2032***	0.0407***	0.0534*
							(2.6087)	(2.9476)	(1.9141)
straegic			0.0950**			0.1351***			0.1077**
			(2.1580)			(3.1053)			(2.3911)
Size	0.8716***	−0.0059	0.4356***	0.8728***	−0.0056	0.4349***	0.8891***	−0.0061	0.4327***
	(12.3555)	(−0.4237)	(27.5375)	(12.3640)	(−0.4034)	(27.4504)	(12.5111)	(−0.4414)	(26.5532)
Lev	3.1077***	−0.0206	0.9338***	3.1110***	−0.0205	0.9629***	3.1238***	−0.0256	0.9969***
	(9.0483)	(−0.2752)	(9.9398)	(9.0584)	(−0.2747)	(10.1810)	(9.0609)	(−0.3433)	(9.5036)
ROA	−0.8813	−1.0942***	3.6684***	−0.9300	−1.1009***	3.7448***	−1.0679	−1.0926***	3.6758***
	(−1.0574)	(−4.8689)	(12.3852)	(−1.1104)	(−4.8861)	(12.5929)	(−1.2886)	(−4.9695)	(12.6717)
Growth	−0.2247**	−0.0021	−0.0660***	−0.2221**	−0.0015	−0.0684***	−0.1830**	−0.0023	−0.0750***
	(−2.5306)	(−0.1667)	(−2.9644)	(−2.5064)	(−0.1202)	(−3.0829)	(−2.0450)	(−0.1768)	(−3.0647)
Cashflow	4.8695***	0.2378	−0.3243	4.8817***	0.2387	−0.3218	4.9203***	0.2499	−0.2525
	(5.0659)	(1.5510)	(−1.3631)	(5.0712)	(1.5550)	(−1.3658)	(5.1140)	(1.6410)	(−0.9924)
Age	1.4052***	−0.0905***	0.0574	1.4132***	−0.0894***	0.0507	1.4551***	−0.0927***	0.1166*
	(6.4222)	(−2.7665)	(1.1763)	(6.4980)	(−2.7320)	(1.0452)	(6.5744)	(−2.8221)	(1.7822)
_cons	−18.1459***	1.2622***	−4.0850***	−18.1931***	1.2557***	−4.1169***	−18.3040***	1.3164***	−4.1213***
	(−10.8954)	(3.9572)	(−9.8060)	(−10.9042)	(3.9284)	(−9.9679)	(−10.8287)	(4.1444)	(−9.5921)
Year fe	Yes	Yes	Yes	Yes	Yes	Yes	Yes	Yes	Yes
industry fe	Yes	Yes	Yes	Yes	Yes	Yes	Yes	Yes	Yes
N	1670	1670	1670	1670	1670	1670	1670	1670	1670
adj. R2	0.2500	0.084	0.542	0.2500	0.084	0.548	0.2415	0.087	0.510

**Table 5:** Mechanism test: Resource allocation efficiency.

Variable	(1)	(2)	(3)	(4)	(5)	(6)	(7)	(8)	(9)
	TFP	Ineff	TFP	TFP	Ineff	TFP	TFP	Ineff	TFP
DA	0.2821***	0.0084***	0.0802***						
	(5.2341)	(3.9869)	(4.7608)						
ODA				0.2849***	0.0082***	0.0772***			
				(5.1733)	(3.9061)	(4.4887)			
DDA							0.2032***	0.0091***	0.0397*
							(2.6087)	(2.7863)	(1.7158)
Ineff			0.4400**			0.4377**			0.2774*
			(2.2360)			(2.2175)			(1.7142)
Size	0.8716***	−0.0013	0.4183***	0.8728***	−0.0012	0.4188***	0.8891***	−0.0011	0.4243***
	(12.3555)	(−0.3503)	(20.3775)	(12.3640)	(−0.3293)	(20.3388)	(12.5111)	(−0.2966)	(24.6827)
Lev	3.1077***	0.0335**	0.9546***	3.1110***	0.0336**	0.9502***	3.1238***	0.0324**	1.0239***
	(9.0483)	(2.3475)	(7.1169)	(9.0584)	(2.3492)	(7.0642)	(9.0609)	(2.2738)	(8.8585)
ROA	−0.8813	−0.0573	3.8752***	−0.9300	−0.0588	3.8540***	−1.0679	−0.0616*	4.1242***
	(−1.0574)	(−1.5380)	(7.3336)	(−1.1104)	(−1.5757)	(7.2443)	(−1.2886)	(−1.6636)	(8.4383)
Growth	−0.2247**	−0.0081**	−0.0358	−0.2221**	−0.0080**	−0.0339	−0.1830**	−0.0073**	−0.0415*
	(−2.5306)	(−2.2083)	(−1.3572)	(−2.5064)	(−2.1833)	(−1.2863)	(−2.0450)	(−1.9901)	(−1.7966)
Cashflow	4.8695***	0.1338***	0.0847	4.8817***	0.1338***	0.0765	4.9203***	0.1373***	−0.0916
	(5.0659)	(3.3815)	(0.2315)	(5.0712)	(3.3810)	(0.2086)	(5.1140)	(3.4524)	(−0.2557)
Age	1.4052***	0.0379***	0.0104	1.4132***	0.0383***	0.0135	1.4551***	0.0384***	0.0392
	(6.4222)	(4.5678)	(0.1523)	(6.4980)	(4.6118)	(0.1988)	(6.5744)	(4.6093)	(0.8086)
_cons	−18.1459***	0.1333	−3.6227***	−18.1931***	0.1310	−3.6406***	−18.3040***	0.1423	−3.7250***
	(−10.8954)	(1.3238)	(−7.6332)	(−10.9042)	(1.2992)	(−7.6632)	(−10.8287)	(1.4132)	(−8.0796)
Year fe	Yes	Yes	Yes	Yes	Yes	Yes	Yes	Yes	Yes
industry fe	Yes	Yes	Yes	Yes	Yes	Yes	Yes	Yes	Yes
N	1670	1670	1670	1670	1670	1670	1670	1670	1670
adj. R2	0.2500	0.131	0.492	0.2500	0.131	0.491	0.2415	0.129	0.564

First, we tested the total effect of DA on TFP. As shown in Columns (1), (4), and (7) of Tables 4 and 5, Hypothesis H1 is further supported by the empirical results.

Second, we examined the effects of DA on the mediating variables (Strategy and Ineff). Table 4, Columns (2), (5), and (8) present the effects of data assetization on strategic distinctiveness. The regression coefficients for DA, ODA, and DDA are positive and statistically significant at the 10%, 5%, and 10% levels, respectively, indicating that higher levels of data assetization are associated with increased strategic distinctiveness. Notably, DDA exhibits the largest coefficient (0.0407), suggesting that transactional data assets—being direct manifestations of value creation—are more readily identifiable and quantifiable, thus having a more pronounced impact in the context of strategic differentiation. Table 5, Columns ()2), (5), and (8) report the influence of data assetization on resource allocation efficiency. The coefficient for DA is 0.0084 and significant at the 10% level, indicating that enterprise data assetization contributes to improved resource allocation efficiency.

Finally, we tested the effects of the mediating variables on TFP and estimated the confidence intervals of the mediating effects using the Bootstrap method to assess the significance of the mediating effects. Columns (3), (6), and (9) of Table 4 show that the regression coefficients for DA, ODA, and DDA are all positive and statistically significant at the 10%, 10%, and 1% levels, respectively, indicating that data assets—both self-used and transactional—can enhance the market insight and resource reconfiguration capabilities of SRDI SMEs. By strengthening internal competitiveness and increasing strategic differentiation, firms can advance their high-quality development, thereby supporting Hypothesis 2. Notably, the coefficient for DDA is significant only at the 1% level, suggesting that in actual enterprise operations, self-used data assets—as the “cause” of high-quality development—play a more prominent role in driving sustainable competitive advantage. Similarly, as shown in Columns (3), (6), and (9) of Table 5, the coefficients for DA, ODA, and DDA remain positive and significant at the 10%, 10%, and 1% levels, respectively, indicating that data assets enable SRI enterprises to overcome the “resource curse” and transcend traditional constraints on resource allocation. Improved resource allocation efficiency contributes to the high-quality development of these enterprises, effectively empowering their long-term growth. The results for transactional data assets support Hypothesis 3. In particular, while DDA is significant at the 1% level, this suggests that compared to transactional data assets, which reflect past outcomes (“what has happened”), self-used data assets facilitate optimal resource allocation through computational analysis and are thus more critical in enhancing efficiency.

### 4.3. Robustness test

#### 4.3.1. Perform the measurement by interchanging the dependent variable and the explanatory variable.

(1) To address the potential endogeneity issue, we replaced the dependent variable for measurement. Given the sufficiently long time span of the sample, the generalized method of moments (GMM) can be employed to mitigate the endogeneity problem. Consequently, this study utilized the firm-level total factor productivity (TFP-FE), estimated using the fixed effects (FE) model, as an alternative variable. This substitution aims to reduce the potential bias in empirical results caused by measurement errors. Further analysis was conducted to explore the impact of data assetization on the high-quality development of SRDI enterprises. As shown in Table 6, the regression coefficients of DA, ODA, and DDA are all significantly positive at the 1% level, which aligns with the benchmark regression results. These findings confirm the robustness of the conclusion that data assetization promotes the high-quality development of enterprises.

**Table 6:** Test results of replacing the explained variable.

Variable	(1)	(2)	(3)	(4)
	TFP-FE	TFP-FE	TFP-FE	TFP-LP
**DA**	0.0596***			
	(3.7163)			
**ODA**		0.0598***		
		(3.4474)		
**DDA**			0.0526***	
			(2.7382)	
**Data_Asset**				0.1383***
				(4.6234)
**Size**	0.7691***	0.7696***	0.7710***	0.3273***
	(49.1550)	(49.1214)	(49.6947)	(10.5325)
**Lev**	1.3050***	1.3056***	1.2933***	0.7869***
	(13.1466)	(13.1219)	(13.1691)	(6.1542)
**ROA**	3.3371***	3.3251***	3.2964***	2.9424***
	(11.2334)	(11.2082)	(11.1599)	(7.1731)
**Growth**	−0.0949***	−0.0942***	−0.0871***	−0.0390*
	(−5.0911)	(−5.0673)	(−4.4856)	(−1.6727)
**CashFlow**	0.4189*	0.4196*	0.4320*	0.0960
	(1.7487)	(1.7434)	(1.8334)	(0.2996)
**Age**	0.1222***	0.1240***	0.1293***	0.0574
	(2.7809)	(2.8222)	(2.8565)	(1.0933)
**_cons**	−7.7341***	−7.7504***	−7.6950***	−4.5283***
	(−19.6082)	(−19.6949)	(−19.5851)	(−9.6827)
***N***	1630	1630	1630	1670
**adj. *R*2**	0.7420	0.7419	0.7395	0.443

(2) To assess the robustness of our findings, we re-specify the explanatory variable following the methodology of Li et al. [29]. Specifically, firm-level data assets (Data_Asset) are measured as the natural logarithm of market value after deducting fixed assets, financial assets, and intangible assets, serving as an alternative to the original data assetization (DA) measure. Market value is calculated as the sum of the book value of total liabilities and the market value of equity. Intangible assets include both financial assets and narrowly defined accounting intangibles. As reported in Column (4) of Table 6, the regression coefficient on Data_Asset remains positive and statistically significant at the 10% level, consistent with the baseline estimates. This confirms the robustness of Hypothesis 1 under alternative variable specifications.

#### 4.3.2. Add control variables and conduct sub-sample regressions.

(1) Corporate governance factors not only significantly influence the effectiveness of enterprise data assets but also act as critical drivers for SRDI SMEs to achieve high-quality development. Due to the substantial number of missing values in corporate governance variables, which could introduce sample selection bias, this robustness test incorporates key governance variables such as dual leadership (Dual), ownership type (SOE), equity concentration (Top10), board independence (Indep), and audit opinion (Opinion) into the regression analysis. The results in Columns (1), (2), and (3) of Table 7 indicate that the regression coefficients of DA and ODA are significantly positive at the 1% significance level, while the coefficient of DDA is significantly positive at the 5% level. These findings confirm the validity of the core conclusion.

**Table 7:** Results of Regression Tests with Additional Control Variables and Sub-samples.

Variable	(1)	(2)	(3)	(4)	(5)	(6)
	TFP	TFP	TFP	TFP	TFP	TFP
**DA**	0.0430***			0.0430***		
	(2.8234)			(2.8234)		
**ODA**		0.0450***			0.0450***	
		(2.8353)			(2.8353)	
**DDA**			0.0725**			0.0725**
			(2.4225)			(2.4225)
**Size**	0.4254***	0.4256***	0.4154***	0.4254***	0.4256***	0.4154***
	(21.5047)	(21.5274)	(19.3717)	(21.5047)	(21.5274)	(19.3717)
**Lev**	0.9738***	0.9751***	1.0734***	0.9738***	0.9751***	1.0734***
	(7.9699)	(7.9802)	(7.6891)	(7.9699)	(7.9802)	(7.6891)
**ROA**	3.4948***	3.4886***	3.7446***	3.4948***	3.4886***	3.7446***
	(8.6079)	(8.6040)	(8.3652)	(8.6079)	(8.6040)	(8.3652)
**Growth**	−0.0648***	−0.0647***	−0.0701***	−0.0648***	−0.0647***	−0.0701***
	(−2.6338)	(−2.6254)	(−2.7719)	(−2.6338)	(−2.6254)	(−2.7719)
**Cashflow**	0.0987	0.0990	0.1297	0.0987	0.0990	0.1297
	(0.3314)	(0.3315)	(0.4147)	(0.3314)	(0.3315)	(0.4147)
**Age**	0.0530	0.0545	0.0591	0.0530	0.0545	0.0591
	(0.7877)	(0.8098)	(0.8579)	(0.7877)	(0.8098)	(0.8579)
**Dual**	−0.0265	−0.0268	−0.0432	−0.0265	−0.0268	−0.0432
	(−0.8854)	(−0.8913)	(−1.3860)	(−0.8854)	(−0.8913)	(−1.3860)
**SOE**	0.0521	0.0516	0.0578	0.0521	0.0516	0.0578
	(1.4075)	(1.3975)	(1.5407)	(1.4075)	(1.3975)	(1.5407)
**Top10**	−0.0004	−0.0004	−0.0009	−0.0004	−0.0004	−0.0009
	(−0.3401)	(−0.3172)	(−0.6233)	(−0.3401)	(−0.3172)	(−0.6233)
**Indep**	−0.4071	−0.4159	−0.4919	−0.4071	−0.4159	−0.4919
	(−1.2092)	(−1.2340)	(−1.4832)	(−1.2092)	(−1.2340)	(−1.4832)
**Opinion**	0.0566	0.0565	0.1071	0.0566	0.0565	0.1071
	(0.3524)	(0.3522)	(0.6669)	(0.3524)	(0.3522)	(0.6669)
**_cons**	−3.4885***	−3.4987***	−3.2356***	−3.4885***	−3.4987***	−3.2356***
	(−6.5860)	(−6.6021)	(−5.6365)	(−6.5860)	(−6.6021)	(−5.6365)
***N***	1586	1586	1586	1586	1586	1586
**adj. *R*2**	0.4486	0.4487	0.4395	0.4486	0.4487	0.4395

(2) At the national level, the promotion of the big data strategy and the establishment of supporting regulations have driven enterprises to accelerate the transformation of data resources into core strategic assets. Marked by the release of China’s 13th Five-Year Plan Outline and the Cybersecurity Law in 2016, a systematic framework for the market-oriented development of data elements was established. The former, through the implementation of the national big data strategy, explicitly called for the establishment of a data sharing and openness mechanism as well as the deepening of data value exploration. The latter, while reinforcing the security baseline, provided institutional safeguards from a legal perspective to ensure the compliant circulation and efficient utilization of data elements. Following prior research practices, samples before 2016 were excluded, and the model was re-estimated. The results in Columns (4), (5), and (6) of Table 7 show that the regression coefficients of DA, ODA, and DDA remain significantly positive at the 1%, 1%, and 5% levels, respectively. These findings support the robustness of the main hypothesis [28].

#### 4.3.3. Eliminate the influence of possibilities.

(1) Excluding the influence of Chinese government policies. Following He et al. [24], controlling for government policy effects has become a widely adopted approach in the existing literature, and this study employs the same strategy to address potential confounding influences. The qualification certification of SRDI enterprises is inherently policy-driven, which may lead firms to simultaneously benefit from both data assetization and direct governmental support. To disentangle the impact of data assetization on high-quality development from that of Chinese government subsidies, we include the government subsidy variable (Sasset) in the regression model. As shown in Column (1) of Table 8, DA remains significantly positive at the 5% significance level, confirming that the influence of government subsidies has been excluded. These findings demonstrate that data assetization plays a core supporting role in the high-quality development path of SRDI SMEs, thereby validating Hypothesis 1.
(2) Adding the digital transformation variable. Prior studies have highlighted that digital transformation can significantly promote the high-quality development of enterprises [29]. To address the potential influence of digital transformation on the conclusions, this paper incorporates the digital transformation variable as a control factor into the benchmark regression model, following previous research practices [30]. The results in Columns (2), (3), and (4) of Table 8 indicate that the regression coefficient of DA remains significantly positive at the 1%, 10%, and 5% significance levels, respectively. This confirms that even after controlling for digital transformation, the positive impact of data assetization on the high-quality development of SRDI SMEs remains robust.

**Table 8:** Regression results excluding the impact of other possibilities.

	(1)	(2)	(3)	(4)
	TFP	TFP	TFP	TFP
**DA**	0.0304**	0.0481***	0.0298*	0.0336**
	(1.9698)	(3.2441)	(1.6583)	(2.0843)
**DCG1**		0.1091		
		(1.1679)		
**DCG2**			0.0015**	
			(2.0915)	
**DCG3**				1.2789*
				(1.8033)
**Sasset**	0.0000			
	(0.9977)			
**Size**	0.4503***	0.4479***	0.4398***	0.4430***
	(21.1900)	(24.5062)	(25.2909)	(25.1898)
**Lev**	0.8861***	0.8840***	0.8723***	0.8599***
	(7.5106)	(7.8954)	(7.8886)	(7.8275)
**ROA**	3.5082***	3.5414***	3.5481***	3.5342***
	(9.7027)	(9.6562)	(9.6613)	(9.6357)
**Growth**	−0.0523**	−0.0560**	−0.0584**	−0.0578**
	(−2.0442)	(−2.2848)	(−2.3852)	(−2.3573)
**CashFlow**	−0.0644	−0.0084	0.0329	0.0014
	(−0.2202)	(−0.0288)	(0.1152)	(0.0048)
**Age**	0.0846	0.0554	0.0587	0.0617
	(1.3161)	(1.1231)	(1.1927)	(1.2460)
**_cons**	−4.2724***	−4.0843***	−3.8996***	−3.9615***
	(−8.9884)	(−9.7663)	(−9.7988)	(−9.8487)
***N***	1657	1639	1639	1639
**adj. *R*2**	0.4445	0.4776	0.4807	0.4782

### 4.4. Endogeneity treatment

#### 4.4.1. Instrumental variable method.

To address the endogeneity issue arising from bidirectional causality, this paper adopts the two-stage least squares (2SLS) method to mitigate its potential influence on the regression analysis. We construct an instrumental variable (DAave) defined as the average level of data assets within the same industry and time period, excluding the firm’s own observation. From a statistical standpoint, firm-level indicators are inherently correlated with the corresponding industry-level mean values, reflecting systematic within-industry patterns. Furthermore, variance inflation factor (VIF) tests show that all VIF values in Table 9 are below 10 and the reciprocal values (1/VIF) exceed 0.1, confirming the absence of severe multicollinearity among the model variables. Therefore, the industry-level average of data assets is unlikely to exert a direct causal effect on enterprise high-quality development, satisfying the exclusion restriction required for valid instrumental variable selection. The regression results presented in Table 10 indicate that the coefficient of DAave is significantly positive at the 1% significance level. Additionally, the Kleibergen-Paap rk Wald F statistic exceeds 16.38, confirming that the selected instrumental variable is not weak. In the second-stage estimation, the regression coefficient of data assetization remains significantly positive at the 1% confidence level, demonstrating that the research conclusion remains robust after addressing the endogeneity issue caused by bidirectional causality.

**Table 9:** The variance inflation factor (VIF) test results.

Variable	VIF	1/VIF
**DAave**	1.090	0.915
**Size**	1.170	0.857
**Lev**	1.360	0.737
**ROA**	1.540	0.648
**Growth**	1.060	0.945
**CashFlow**	1.350	0.741
**Age**	1.030	0.968
**Mean VIF**	1.230	

**Table 10:** Results of instrumental variable method.

variable	TFP	CP
	The first stage of return	The second stage of return
**DA**	—	0.071***
	—	(0.027)
**Control variable**	control	control
**Year/Industry**	control	control
**instrumental variable (ROA)**	0.711***	—
	(0.035)	—
**sample size**	1670	1670
**Kleibergen-Paap rk LM** **P-value**	0.000	
**Kleibergen-Paap rk** **Wald F statistic**	420.728 (16.38)	

#### 4.4.2. Propensity score matching (psm) analysis.

**Fig 1:**
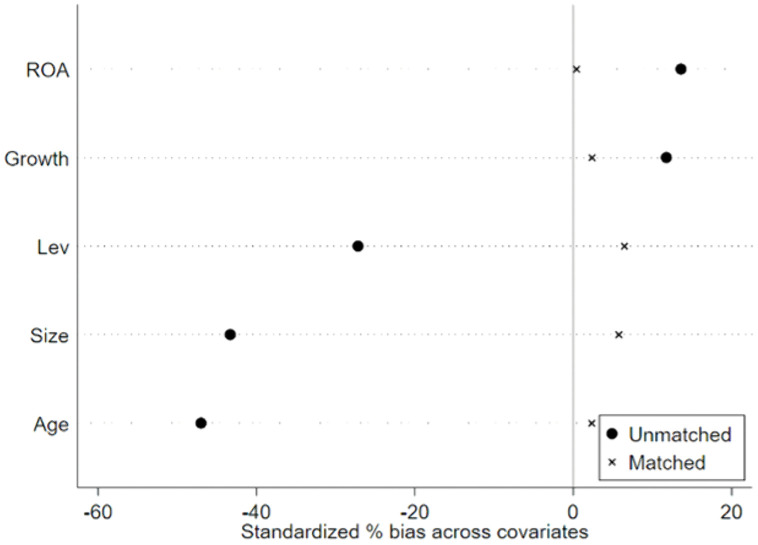
Standardized deviation of variables.

**Fig 2:**
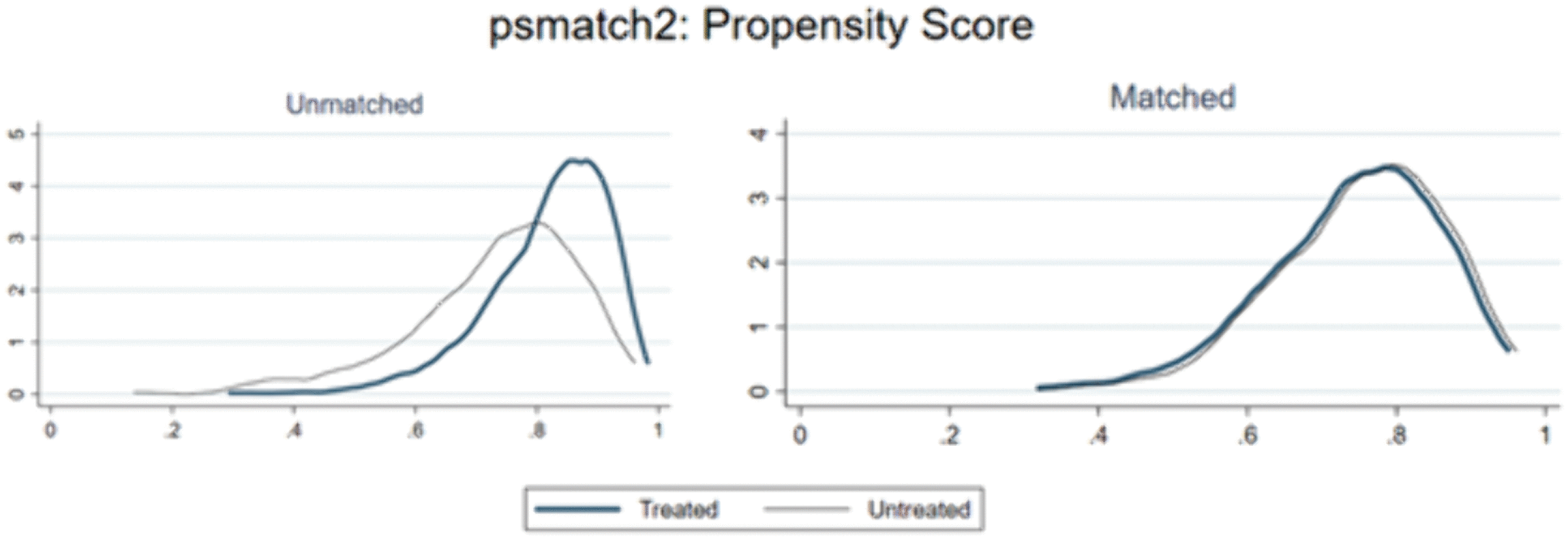
Kernel density plot of propensity scores.

To further mitigate the potential interference of external macroeconomic factors on the empirical results, this study applies the propensity score matching (PSM) method to process the samples. Given that in 2016, China’s Ministry of Industry and Information Technology released the “Big Data Industry Development Plan (2016–2020)”, which served as a typical exogenous shock event to drive the innovative development of industrial big data, the policy time point for the difference-in-differences (DID) model is set at 2016. During the matching procedure, drawing upon prior research [31], control variables are utilized as matching covariates, and a 1:1 nearest neighbor matching approach with a caliper width of 0.01 is implemented. The effectiveness of the matching is validated through balance tests. As shown in Table 11, after matching, the differences in all covariates between groups become statistically insignificant, and all t-tests fail to reject the null hypothesis of no systematic differences between the treatment and control groups, thereby confirming the successful achievement of balance. Fig 1 illustrates the reduction in standardized biases of variables post-matching, indicating a significant decrease across all variables and thus validating the robustness of the matching process. Furthermore, the kernel density distribution of propensity scores presented in Fig 2 demonstrates enhanced overlap between the experimental and control groups after matching, satisfying the common support assumption. These findings collectively suggest that, after accounting for sample selection bias, data assetization continues to empower the high-quality development of SRDI enterprises, reinforcing the robustness of the conclusions drawn in this paper.

**Table 11:** Results of balance test for propensity score matching.

Variable name		Mean value		Standard deviation (%)	Percentage reduction in standard deviation (%)	t-statistic	t-test
		Treatment group	Control group				p > t
**Size**	Unmatched	21.467	21.83	−43.3	86.8	−7.45	0.000
	match	21.805	21.757	5.7		0.72	0.474
**Lev**	Unmatched	0.32469	0.37464	−27.2	76.3	−4.44	0.000
	match	0.38125	0.36942	6.4		0.80	0.426
**ROA**	Unmatched	0.03799	0.02815	13.6	97.1	2.26	0.024
	match	0.03092	0.03064	0.4		0.05	0.962
**Growth**	Unmatched	0.25604	0.18376	11.8	80.0	1.81	0.071
	match	0.21376	0.20002	2.3		0.31	0.758
**Age**	Unmatched	2.937	3.0631	−47.0	95.1	−7.40	0.000
	match	3.0594	3.0532	2.3		0.31	0.755

#### 4.4.3. Fixed effects control test.

This study further incorporates the individual fixed effects of enterprises and simultaneously controls for the interaction fixed effects between industry and year, as well as between province and year, in the regression analysis. As shown in Table 12, the regression coefficients of DA, ODA, and DDA are positively significant at the 1%, 1%, and 10% significance levels, respectively, confirming the robustness of the core conclusion.

**Table 12:** Results of endogeneity treatment.

	(1)	(2)	(3)
	TFP	TFP	TFP
**DA**	0.5835***		
	(2.8695)		
**ODA**		0.5676***	
		(2.7342)	
**DDA**			0.6276*
			(1.8926)
**Size**	1.0825*	1.1075*	1.4460**
	(1.7788)	(1.8379)	(2.3686)
**Lev**	6.0959***	5.9844***	7.3768***
	(3.4550)	(3.3707)	(4.1193)
**ROA**	3.3527	3.1660	4.7964
	(1.1169)	(1.0588)	(1.4521)
**Growth**	0.0241	0.0317	−0.3793
	(0.0915)	(0.1198)	(−1.1908)
**Cashflow**	1.8930	2.0138	4.0552
	(0.8179)	(0.8727)	(1.6414)
**Age**	6.6889*	6.3817*	10.1792*
	(1.9063)	(1.8340)	(1.7763)
**_cons**	−40.5600**	−40.0789**	−58.8304***
	(−2.4692)	(−2.4615)	(−2.8270)
***N***	1670	1670	1670
**adj. *R*2**	0.2115	0.2091	0.2233

### 4.5. Further analysis: Heterogeneity test based on the TOE framework

The TOE framework was proposed by Tornatzky and Fleischer in 1990, namely the “Technology–Organization–Environment” analysis framework (TOE framework). This theoretical framework focuses on the interactive relationship among technology, organization, and environment, and is a theoretical model used to analyze the reasons for the behavioral decisions made by enterprises or organizations in different contexts [32]. Against the backdrop of the digital transformation era, the high-quality development and data assetization process of SRDI SMEs are deeply interrelated, and the interaction among the three dimensions of technology, organization, and environment significantly shapes this correlation mechanism. As the business environment and management complexity of enterprises increase, the interdependence among technology, organization, and environment elements intensifies, jointly influencing the path through which data assetization affects the financing constraints of SRDI SMEs. The TOE analytical framework enables a systematic examination of the heterogeneous effects of data assetization on the high-quality development of SRDI SMEs from multiple dimensions.

#### 4.5.1. Technical competence.

Enterprise innovation data integrates a substantial amount of technical information and serves as a tangible representation of its technological capabilities. The new data, models, and algorithms generated during the innovation process can be considered part of an enterprise’s data assets. These assets can either be commercialized or utilized for internal optimization, thereby driving the high-quality development of enterprises. Furthermore, as the core driver of sustainable enterprise development, market entities with stronger innovation capabilities are better positioned to respond swiftly to market changes and technological advancements. By maintaining competitive advantages, they enhance enterprise value and promote high-quality development. To comprehensively evaluate the impact of data assetization on the innovation capabilities of SRDI SMEs, this study constructs an enterprise innovation capability evaluation system from three dimensions: innovation input (RD), innovation output (Patent), and innovation efficiency (InnoEff) Specifically, RD is measured by the proportion of R&D expenditure to total current assets; Patent is represented by the natural logarithm of the total number of invention patents, utility model patents, and design patents applied for plus one; InnoEff is defined as the ratio of innovation output to the natural logarithm of R&D expenditure plus one. The regression results of the interaction terms are presented in Columns (1), (2), and (3) of Table 13. The regression coefficients of the interaction terms between DA and RD, Patent, and InnoEff (DA*RD, DA*Patent, DA*InnoEff) are all significantly positive at the 1% significance level. This indicates that when enterprises possess stronger technological capabilities, the driving effect of data assets on the high-quality development of SRDI SMEs becomes significantly enhanced. This is because enterprises with stronger digital innovation capabilities can more efficiently transform data resources into assets and apply them, further strengthening the enabling role of data assetization in high-quality development through enriched information resource reserves.

**Table 13:** Further analysis of regression results.

	(1)	(2)	(3)	(4)	(5)	(6)	(7)	(8)	(9)
	TFP	TFP	TFP	TFP	TFP	TFP	TFP	TFP	TFP
**DA**	0.0826***	0.1194***	0.1149***	0.0838**	0.0437**	0.4067**	0.3340**	0.2813*	0.1372**
	(4.2815)	(3.4848)	(3.3680)	(2.3405)	(2.2117)	(2.1927)	(2.2492)	(1.6630)	(2.1563)
**RD**	7.6751***								
	(7.1355)								
DA*RD	1.8484***								
	(4.1990)								
**Patent**		0.0385*							
		(1.7194)							
DA*Patent		0.0310***							
		(2.8952)							
**InnoEff**			0.0873						
			(0.2172)						
DA*InnoEff			0.5339***						
			(2.8411)						
**CUS**				0.0024					
				(1.4848)					
DA*CUS				0.0020**					
				(2.0310)					
**CUSHHI**					0.0055				
					(1.5576)				
DA*CUSHHI					0.0056**				
					(2.1726)				
**DigFin**						0.0040***			
						(3.7688)			
DA*DigFin						0.0013**			
						(2.1922)			
**DigFinCov**							0.0028***		
							(3.2537)		
DA*DigFinCov							0.0010**		
							(2.2498)		
**DigFinUse**								0.0038***	
								(3.6975)	
DA*DigFinUse								0.0010*	
								(1.6956)	
**Fintech**									0.0850***
									(3.0978)
DA*Fintech									0.0367**
									(2.4450)
**Size**	0.4234***	0.4370***	0.4360***	0.4309***	0.4320***	0.4321***	0.4327***	0.4303***	0.4348***
	(19.1961)	(18.7561)	(19.1471)	(22.0768)	(22.5236)	(16.9616)	(16.9932)	(16.7790)	(18.8275)
**Lev**	0.9802***	0.9680***	0.9685***	0.9554***	0.9632***	1.2041***	1.1987***	1.2129***	0.9136***
	(7.1758)	(7.1524)	(7.1322)	(7.0467)	(7.1451)	(7.1645)	(7.1284)	(7.1669)	(6.1493)
**ROA**	3.4635***	3.5552***	3.5556***	3.3779***	3.4179***	3.3516***	3.3513***	3.3661***	2.9820***
	(8.8934)	(9.1105)	(9.1279)	(8.8080)	(8.9690)	(6.7513)	(6.7651)	(6.6751)	(6.7282)
**Growth**	−0.0540**	−0.0633**	−0.0624**	−0.0563**	−0.0540**	−0.0437	−0.0434	−0.0437	−0.0536**
	(−2.1939)	(−2.5212)	(−2.4877)	(−2.0053)	(−1.9738)	(−1.3844)	(−1.3710)	(−1.3917)	(−2.1264)
**CashFlow**	−0.1316	−0.1994	−0.2006	0.0639	0.0588	0.5613	0.5629	0.5452	0.3590
	(−0.5150)	(−0.7810)	(−0.7871)	(0.2128)	(0.1952)	(1.5279)	(1.5285)	(1.4770)	(1.1368)
**Age**	0.1089*	0.0788	0.0792	0.0821	0.0763	0.1847**	0.1834**	0.1853**	0.0697
	(1.7357)	(1.2459)	(1.2524)	(1.2930)	(1.2038)	(2.2377)	(2.2162)	(2.2565)	(1.0804)
**_cons**	−3.8308***	−4.0662***	−4.0197***	−3.8450***	−3.8233***	−5.1990***	−4.9406***	−4.9910***	−3.9911***
	(−8.0529)	(−8.0578)	(−8.1751)	(−8.4409)	(−8.5651)	(−7.5613)	(−7.6084)	(−7.3227)	(−7.4626)
***N***	1649	1649	1649	1678	1678	1560	1560	1560	1560
**adj. *R*2**	0.4352	0.4254	0.4262	0.4125	0.4198	0.3516	0.3509	0.3515	0.3479

#### 4.5.2. Customer relationship.

As a key stakeholder in business relationships, customer relationships play a central role in each link of an organization’s supply chain. On the one hand, strong customer relationships facilitate the acquisition of more accurate and comprehensive customer data, thereby improving data quality. Through ongoing interaction with customers, stable relationships can generate synergistic effects. Enterprises are not only able to better interpret the underlying meaning of data, breaking down “data silos”, but also transform these insights into actionable strategies that enhance the value creation of data assets. On the other hand, from the perspective of social network theory, long-term and stable social network connections are crucial for building a collaborative organizational ecosystem. A deeply embedded customer relationship network can effectively reduce the costs associated with resource acquisition, promote coordinated development, and contribute to achieving high-quality development goals. Studies indicate that using customer concentration (CUS, defined as the ratio of sales to the top five customers relative to annual total sales) and the Herfindahl-Hirschman Index of customer concentration (CUSHHI), higher levels of customer concentration reflect stronger stability and tighter integration within an enterprise’s customer relationship network [33]. The regression results for the interaction terms are presented in Columns (4) and (5) of Table 13. The regression coefficients of the interaction terms between data assetization and CUS (DA*CUS) and CUSHHI (DA*CUSHHI) are both significantly positive at the 5% significance level. This suggests that when customer concentration is high, the driving effect of data assets on the high-quality development of SRDI SMEs is further strengthened. This indicates that close and stable customer relationships can send positive external signals. Such relationships not only accelerate the process of enterprise data assetization but also empower high-quality development by enhancing strategic decision-making capabilities.

#### 4.5.3. The level of digital finance development.

Digital finance, by leveraging diverse cutting-edge technologies, has enabled enterprises to develop efficient data processing and analytical capabilities, thereby optimizing the management and utilization of data resources. Through digital finance tools, enterprises can effectively transform the value of their data assets, empowering the process of data assetization. Moreover, digital finance integrates innovative technologies such as big data, cloud computing, and artificial intelligence into areas like technology finance, green finance, and inclusive finance, promoting the deep integration of finance and technology. This significantly enhances the quality, efficiency, convenience, and market competitiveness of financial services, driving the coordinated development of the digital economy and the real economy, and fostering high-quality economic and enterprise growth. Consequently, in regions with a higher level of digital finance development, SRDI SMEs benefit from a more favorable development environment, enabling them to achieve high-quality development. To measure the environmental dimension more precisely and comprehensively, this paper examines the development levels of regional digital finance and enterprise finance from two perspectives: ① Drawing on Peking University’s digital inclusive finance index system, we select the total index of digital finance development (DigFin) and its sub-dimensions of coverage breadth (DigFinCov) and usage depth (DigFinUse). Based on the registered location of enterprises, the natural logarithm of the municipal-level digital finance development index is used as a proxy variable for the level of digital finance development [34]. ② The level of fintech development (Fintech) is also considered. Following the method of Huang Lei *et al.*, the frequency of fintech-related keywords is extracted from the annual reports of listed companies using machine learning techniques, and the results are logarithmically transformed. The regression results presented in Columns (6), (7), (8), and (9) of Table 13 show that the interaction terms between data assetization and the level of digital finance development—namely DA*DigFin, DA*DigFinCov, DA*DigFinUse, and DA*Fintech—have positive coefficients that are statistically significant at the 5%, 5%, 1%, and 5% levels, respectively. These findings indicate that the higher the regional level of digital finance development and the degree of fintech advancement among listed companies, the stronger the promoting effect of data assetization on the high-quality development of SRDI SMEs. The results confirm that digital finance facilitates the deep integration of financial services with the real economy for SRDI enterprises, providing comprehensive support for enterprise data assetization and empowering these SMEs to achieve their high-quality development goals.

## 5. Conclusions and enlightenment

### 5.1. Research conclusions

The assetization of enterprise data enables enterprises to transition from reliance on traditional production factors to intelligence-driven operations, providing robust support for the high-quality development of “specialized, refined, distinctive, and innovative” (SRDI) small and medium-sized enterprises (SMEs), thereby contributing to the high-quality development of the economy and society. This study uses SRDI SMEs in China from 2013 to 2023 as the research sample, constructs a keyword graph based on enterprise annual report texts to quantify the indicators of enterprise data assetization, and follows the logical path of “establishing benchmark analysis–verifying the mechanism of action–conducting heterogeneity tests” to comprehensively explore how data assetization empowers the high-quality development of SRDI SMEs. The research conclusions are as follows:

(1) For China, data assetization can effectively drive the high-quality development of SRDI SMEs, with both self-utilized and transactional data assets contributing positively to this process. For the global community, although China’s approach to data assetization is grounded in its domestic market, it objectively offers an Eastern model that can inform the high-quality development of innovative SMEs worldwide. Given the growing urgency for international SMEs to enhance their core competitiveness, data assetization has emerged as a strategic imperative. This article outlines potential pathways to support enterprise-level high-quality development through effective data utilization;
(2) For China, data assetization can propel the high-quality development of SRDI SMEs through a dual-driver mechanism—enhancing strategic differentiation and improving resource allocation efficiency. For the global community, China’s model and practical experience in advancing data assetization among such SMEs provide a viable reference framework. International innovative SMEs can leverage this synergistic model of strategic differentiation and resource allocation to expand their strategic thinking, customizing its implementation according to their respective national contexts, including legal systems, financial environments, and other institutional realities;
(3) For China, analysis within the TOE framework reveals that enterprise innovation capacity, customer relationships, and the development level of regional and firm-level digital finance significantly enhance the impact of data assetization on high-quality development. For the global community, China’s experience has empirically validated the applicability of the TOE framework to data assetization, demonstrating that enterprise high-quality development also necessitates transformations in technology, organizational management, and business models. International innovative SMEs should adopt a strategy of “learning from but not replicating” Chinese practices, adapting them to local contexts to strengthen their competitiveness in the global digital economy.

The findings of this study enrich the body of knowledge on data assets at the micro level and provide empirical evidence and practical insights for global efforts to advance the digital transformation and high-quality development of SRDI SMEs. China’s experience in data assetization offers a valuable reference for international firms navigating similar developmental challenges. For global innovative SMEs, the critical factor lies in strengthening their foundational data capabilities, dynamically adapting to evolving regulatory frameworks within a global context, and actively pursuing their own pathways toward high-quality development.

### 5.2. Policy recommendations

Based on the above conclusions, this article proposes the following recommendations:

(1) From the perspective of regulatory authorities, including government agencies, efforts should be directed toward optimizing the environment for data assetization, establishing a foundational institutional framework for data elements, and fostering an open, collaborative, secure, and well-regulated data ecosystem. Specifically:

To optimize the policy environment and institutional framework for data assetization, a three-tier coordinated strategic guidance system—centered on “central planning–local innovation–enterprise practice”—should be established. An accounting standards framework that integrates iterative refinement of accounting principles with standardized information disclosure requirements should be developed. A legal safeguard system encompassing well-defined data ownership arrangements and strengthened intellectual property protection should be built. Additionally, a data factor market system grounded in unified market rules and diversified transaction models should be advanced. Collectively, these initiatives provide a robust enabling environment and clear policy direction for the data assetization of SRDI enterprises;

Promote data asset empowerment platforms and trusted data circulation. A government-led data asset trading platform should be established, complemented by sector-specific data resource pools designed to integrate data from across the industrial chain—particularly from upstream suppliers and downstream enterprises. By leveraging blockchain technology, robust mechanisms for data rights authentication and secure, trustworthy sharing can be implemented, thereby facilitating market-driven circulation and transaction of data assets and expanding data access for SRDI enterprises;

Strengthen the fiscal and tax incentive framework for data assetization. A dedicated digital transformation fund should be established, accompanied by a dual-track policy approach that simultaneously enhances incentives (“adding impetus”) and reduces financial burdens (“reducing burden”). Eligible SRDI enterprises should receive targeted fiscal subsidies, preferential tax treatment for data transactions, accelerated depreciation allowances for data-related assets, and an increased super-deduction rate for data research and development expenditures. These measures collectively lower the cost of data assetization and support enterprises in advancing toward high-quality development.

(2) From the perspective of financial institutions such as banks, there is a strategic imperative to develop tailored data asset products that address the core financing and operational needs of SRDI SMEs. This involves systematically innovating and enhancing the financial product system for data assets to better support their high-quality development. Specifically:

Banks should develop strategic data asset products by establishing industry-level data standards and integrating blockchain technology to enable end-to-end traceability across the data lifecycle. They should leverage smart contract tools to enhance the management mechanisms for data authorization and usage rights. Through comprehensive data asset analysis, banks can deliver more accurate risk assessment and investment decision support to enterprises, while offering customized services to improve customer satisfaction;

Co-develop an industrial data financial service platform. Banks should collaborate with industrial parks and enterprises to enable intelligent matching between data demand and financial services. Simultaneously, they should introduce a “data safe box” service that offers integrated advisory solutions for data asset transactions, including secure evidence preservation, valuation, and clearing mechanisms.

(3) From the perspective of SRDI SMEs enhancing data assetization capabilities and optimizing resource allocation are essential to realizing tangible, measurable value from data. Specifically, enterprises can advance their data assetization initiatives through a three-phase strategy: “taking stock”, “putting into use”, and “turning into money”.

The “taking stock” phase requires comprehensive data inventory and governance to achieve a clear understanding of organizational data assets. Enterprises should establish cross-functional task forces to identify core data sets, evaluate data quality, verify data provenance, and simultaneously develop standardized rules and procedures to enhance data reliability and consistency.

The “putting into use” phase demands a focus on practical business scenario applications to enable data to “generate economic returns”. Enterprises should advance business digitalization by identifying critical operational processes, developing visual dashboards, quantifying the impact of data utilization, and validating the latent value of data assets.

The “turning into money” phase requires exploring viable assetization pathways to transform data into recognized assets. Enterprises should formalize qualified data resources by recording them on financial statements, engage with big data exchanges to obtain information on data product listing and trading mechanisms, and proactively collaborate with financial institutions to facilitate data-backed pledge financing. These measures will accelerate enterprise data assetization and support the high-quality development of SRDI SMEs.

### 5.3. Research limitations and future research

Currently, research on data assetization remains constrained by several limitations. At the theoretical level, fundamental challenges persist, including the absence of consensus on the definition and attributes of data elements, ambiguous data ownership rights, and the lack of a systematic framework for data value assessment. At the practical level, data assetization encounters multiple obstacles, such as underdeveloped data trading markets, insufficient technological infrastructure and talent reserves, and limited effectiveness in ensuring data security and privacy protection. At the macro-environmental level, institutional and structural barriers remain, including an incomplete policy and regulatory system and significant disparities in development across regions and industries. Therefore, future research should prioritize the following key directions:

① Advancing interdisciplinary integration in data assetization to construct a comprehensive and cohesive theoretical framework; ② Strengthening practice-oriented approaches by developing actionable implementation strategies and toolkits for enterprises, while exploring balanced mechanisms that reconcile data security with privacy protection; ③ Enhancing policy support through the development of industry-specific data assetization pathways and models, and fostering regional coordination and ecosystem building to enable sustainable data-driven transformation.
